# Multiple, but Concerted Cellular Activities of the Human Protein Hap46/BAG-1M and Isoforms

**DOI:** 10.3390/ijms10030906

**Published:** 2009-03-02

**Authors:** Ulrich Gehring

**Keywords:** Apoptosis, DNA binding, hsp70 molecular chaperones, multifunctional proteins, protein folding, transcriptional regulation

## Abstract

The closely related human and murine proteins Hap46/BAG-1M and BAG-1, respectively, were discovered more than a decade ago by molecular cloning techniques. These and the larger isoform Hap50/BAG-1L, as well as shorter isoforms, have the ability to interact with a seemingly unlimited array of proteins of completely unrelated structures. This problem was partially resolved when it was realized that molecular chaperones of the hsp70 heat shock protein family are major primary association partners, binding being mediated by the carboxy terminal BAG-domain and the ATP-binding domain of hsp70 chaperones. The latter, in turn, can associate with an almost unlimited variety of proteins through their substrate-binding domains, so that ternary complexes may result. The protein folding activity of hsp70 chaperones is affected by interactions with Hap46/BAG-1M or isoforms. However, there also exist several proteins which bind to Hap46/BAG-1M and isoforms independent of hsp70 mediation. Moreover, Hap46/BAG-1M and Hap50/BAG-1L, but not the shorter isoforms, can bind to DNA in a sequence-independent manner by making use of positively charged regions close to their amino terminal ends. This is the molecular basis for their effects on transcription which are of major physiological relevance, as discussed here in terms of a model. The related proteins Hap50/BAG-1L and Hap46/BAG-1M may thus serve as molecular links between such diverse bioactivities as regulation of gene expression and protein quality control. These activities are coordinated and synergize in helping cells to cope with conditions of external stress. Moreover, they recently became markers for the aggressiveness of several cancer types.

## 1. The protein family

Two related proteins, BAG-1 and Hap46, were discovered independently in the mid 1990s using molecular genetic methods by three research groups who were interested in quite different biological phenomena. We knew from previous experiments using chemical crosslinking methods with the liganded and activated glucocorticoid receptor, a member of the nuclear receptor superfamily, that this protein is able to form large complexes with other proteins while associating with the genome. Therefore, we set out to search for interaction partners by using immunochemical screening of a human liver cDNA library, since liver is a major target for this class of hormones. We thus cloned a cDNA of 1,322 base pairs which encodes a 274 residue polypeptide [1,2]. The apparent molecular weight is about 46 kDa in SDS polyacrylamide gels, thus the original designation RAP46 for “*receptor-associating protein of 46 kDa apparent molecular mass*” [2]. However, this name was subsequently changed to **Hap46**, *i.e.* “*hsp70/hsc70-associating protein*“ [3] when we realized that hsp70 heat shock proteins are the most prominent interaction partners (see below). In a similar approach, Takayama *et al*. [4] looked for binding partners for the anti-apoptotic protein Bcl-2 in a mouse embryonic expression library and obtained a cDNA clone of 830 base pairs coding for a 219 amino acid sequence. As this sequence produced in transfection experiments inhibitory effects on apoptosis induced by several treatments, the protein was termed “*Bcl-2-associated athanogen*“, in short **BAG-1** [4], with the Greek word *athánatos* meaning “anti-death“. A similar murine cDNA expression library was also used by a third research group [5] who employed the cytoplasmic domain of the plasma membrane receptor for hepatocyte growth factor as interacting component in their cloning approach. They isolated several independent clones of different length, which however contained the same cDNA coding for the BAG-1 protein of about 29 kDa apparent molecular size. These authors further described complex formation not only with the hepatocyte growth factor receptor, but also with that for platelet-derived growth factor. Again, anti-apoptotic effects were observed [5].

At first, it was quite surprising that the above described proteins, human Hap46 and murine BAG-1, are up to 25 % different in size and have distinct amino terminal regions, although they are homologous otherwise. It then turned out that they are but two members of a protein family which comprises several isoforms.

**Figure 1.**
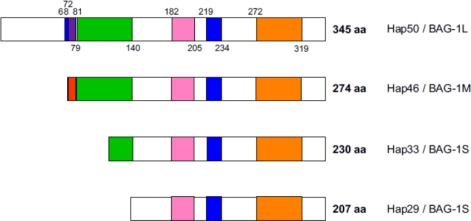
Domain structure of polypeptides.*Blue*: potential nuclear localization signals; *red*: basic DNA-binding domain. The sequence in Hap46/BAG-1M is (1)Met-Lys-Lys-Lys-Thr-Arg-Arg-Arg-Ser-Thr(10). Due to overlap with a potential nuclear localization signal, this appears *purple* in Hap50/BAG-1L; *green*: acidic hexarepeat domain, consensus sequence: Thr-Arg-Ser-Glu-Glu-X; *pink*: ubiquitin-like domain; *orange*: hsp70/hsc70 binding domain or BAG-domain (shown here as originally defined [30,38]).

While human Hap46 of 274 amino acid residues is synthesized from an AUG codon and thus starts out with methionine (*cf.* Figure 1), it became evident upon cloning of 5’-extended cDNAs that a larger isoform exists. This originates from an upstream CUG non-canonical start codon, begins with leucine and contains 345 amino acids [6]. In comparison to Hap46, it is 71 amino acids longer at the amino terminal end, and since the apparent molecular weight is roughly 50 kDa, it has been called **p50**, **Hap50** or **BAG-1L** (L for long). This isoform is present in human and murine cells [6–8]. For the sake of nomenclature, human Hap46 was then renamed **BAG-1M** (M for medium). There also exist shorter human isoforms (*cf.* Figure 1), most importantly the 230 residue **Hap33/BAG-1S** (S for small), as well as the even shorter form **Hap29/BAG-1S** of 207 amino acids [7,8], which originate from AUG codons further downstream. Expression of these isoforms occurs at very different levels in different cell types, however, Hap33/BAG-1S appears to be the most prominent form in the majority of cells under normal conditions and Hap29/BAG-1S is not consistently detected in all cells.

There is no direct correlate to human Hap46/BAG-1M in the mouse since the murine message has a GUG (coding for valine) in the corresponding position instead of AUG. The originally discovered 219 residue murine protein [4] is a somewhat shorter isoform which is produced from an internal AUG start codon [9] and roughly corresponds to human Hap33/BAG-1S. In order to avoid confusions, this protein is called “mouse BAG-1S” in this review. Most significantly, however, the murine and human protein factors are highly homologous.

The message is roughly 1.4 kb long [2] and harbors several translation initiation codons, giving rise to the series of polypeptide chains depicted schematically in Figure 1. The gene coding for these proteins is called “***Bag1***“ and is located on human chromosome 9 in region p12 [10]; it is roughly 10 kb in size and contains seven exons. The promoter covers positions −353 to −54 upstream of the first translational start codon, lacks a TATA box but has a CCAAT box and contains binding sites for several transcription factors, which may be important in regulating the expression [11]. Interestingly, p53 mutants obtained from human tumors are able to upregulate the *Bag1* promoter [11]. Moreover, expression of the *Bag1*gene is modulated by methylation reactions [12].

## 2. Polypeptide domains

Figure 1 shows that all protein isoforms have a common carboxy terminal region, but differ significantly in their amino terminal portions. Consequently, they share the functions mediated by the carboxy terminal part, possibly with individual additional molecular properties otherwise. It thus appears that all isoforms are roughly equivalent in biochemical reactions mediated by the carboxy terminal domain (orange in Figure 1), *i.e.* interactions with hsp70 heat shock proteins (see below).

**Figure 2.**
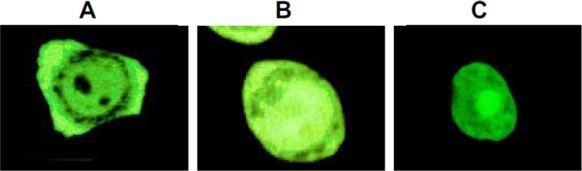
Intracellular distribution of Hap46/BAG-1M and Hap50/BAG-1L. (A and B) HeLa cells were transfected with a construct in which the sequence encoding Hap46/BAG-1M was coupled to that for the green fluorescent protein GFP. In A, cells were maintained at 37°C, in B they were exposed to a 42°C treatment for 2 h. Analysis was by fluorescence microscopy. From ref. [13]. (C) HeLa cells, similarly transfected with a construct coding for Hap50/BAG-1L coupled to GFP, were maintained at 37°C. In contrast to A and B, the cell body and the contours are practically invisible due to the very strong fluorescence originating from the nucleus. From ref. [14].

A striking characteristic of Hap50/BAG-1L is a series of positively charged residues in proximity to the amino terminus, as is typical for nuclear localization signals (blue in Figure 1). However, Hap46/BAG-1M also contains a cluster of basic amino acids right next to its amino terminus. Both of these regions in Hap46/BAG-1M and Hap50/BAG-1L are of significance for interactions with nucleic acids (see below). By contrast, isoforms Hap33/BAG-1S, Hap29/BAG-1S, and mouse BAG-1S do not contain these basic sequences and — as a consequence — are unable to bind to DNA. The potential nuclear localization signal roughly in the middle of the Hap46/BAG-1M sequence (blue in Figure 1) appears to be masked by some as yet unknown mechanism, but apparently becomes activated upon heat shock, since a significant portion of Hap46/BAG-1M accumulates in the nuclear compartment in response to thermal stress (Figure 2, B *vs.* A).

The functional significance of the acidic hexarepeat domain (green in Figure 1) remains rather enigmatic at present, as it is quite variable amongst different isoforms. Deletion of this region did neither destroy transcriptional stimulation by Hap46/BAG-1M [15] nor was the acidic hexarepeat domain required for the inhibition of glucocorticoid-dependent transcription in transfected cells [16]. On the other hand, this domain has been reported responsible for the downregulation of glucocorticoid receptor mediated transactivation by Hap46/BAG-1M [17].

The ubiquitin-like domain (pink in Figure 1) is much shorter than ubiquitin itself, but nevertheless mediates cooperation with the proteasome machinery of protein degradation [18,19]. Conjugation with ubiquitin normally primes proteins for degradation by proteasomes, but Hap46/BAG-1M is relatively long-lived, even though it is ubiquitinylated [20]. The ubiquitin-like domain was found to be required for promoting the apoptotic activity of a synthetic retinoid [21], and prevention of stress-induced growth inhibition by Hap46/BAG-1M and isoforms depended on a conserved lysine residue within this domain [22]. On the other hand, the ubiquitin-like domain was expendable for Hap50/BAG-1L to affect the regulatory activity of androgen receptors [23].

## 3. Hsp70 molecular chaperones as primary interaction partners

Already the discovery of this new protein family disclosed the unusual property that its members interact with several completely unrelated protein structures. Aside from the proteins used in the original interaction screening assays (see above), several additional proteins soon became known to be able to associate with Hap46/BAG-1M and mouse BAG-1S. These were other members of the nuclear receptor family [2,24,25], platelet-derived growth factor receptor [5], Raf-1 protein kinase [26], several unrelated transcription factors [3], Siah-1A, a human homologue of *Drosophila* seven in absentia [27], and proteasomes [18]. However, light came into this puzzling problem when it was realized that primary and major interaction partners are members of the 70 kDa heat shock protein family. When several protein-protein interaction assays were employed to identify direct binding partners for Hap46/BAG-1M and mouse BAG-1S, the only polypeptide of significance was about 70 kDa in size and this was obtained with extracts from very different cells. It was then identified as the mammalian heat shock protein hsp70/hsc70 [3, 28–30]. However, to our great surprise the mammalian endoplasmic form BiP, as well as the hsp70 homologues in *Saccharomyces cerevisiae* and of bacterial and plant origin were found unable to bind to Hap46/BAG-1M. By contrast, hsp70 contained in Sf9 cells from the insect *Spodoptera frugiperda*, used in the baculovirus expression system for producing bait proteins, did interact perfectly well [3].

The stress-regulated form — hsp70 in the strict sense — originates in eukaryotes from several genes whose expression is controlled by heat and other types of stress. However, there also exists a slightly larger, but constitutively expressed counterpart, called hsc70. This very fact certainly points to some basic cellular function: the “chaperoning“ of misfolded or partially unfolded polypeptides. Hsp70s are a large and highly conserved family of *molecular chaperones* which function in quite diverse cellular processes. In particular, they are able to bind a wealth of protein structures, especially if these are partially denatured, *i.e.* if they contain stretches of unfolded or misfolded polypeptides. Besides supporting newly synthesized and misfolded polypeptides to find their correct and biologically active conformations, they also minimize the aggregation of such proteins and they function in translocating proteins through membranes (for reviews, see refs. [31–34]). Moreover, they are important for the assembly of multimeric proteins, as for example in generating the heteromeric forms of steroid hormone receptors [35,36] and of transcriptional complexes, as pointed out below, as well as of several other large protein complexes.

All members of the hsp70 heat shock protein family consist of two major domains which are very different in structure (for reviews, see refs. [31,34,37]). The amino terminal portion of about 45 kDa is build up of two somewhat similar subdomains, I and II, each consisting of several α-helices. Together they form the so-called *ATP-binding domain* with the binding site for nucleotides located in the central cleft between them. The carboxy terminal portion of the hsp70 polypeptide is called the *substrate-binding domain*. It is intricately folded to accept the various partner proteins for binding in a channel within a ß-sandwich structure. Obviously, there is strong functional coupling by conformational changes between the ATP-binding and the substrate-binding domains.

The interaction of Hap46/BAG-1M and all isoforms makes use of the common carboxy terminal region, as has originally been shown by deletion of 47 residues from this end of the polypeptide [30,38]. Since this interaction is a very prominent property of the protein family, this common portion has been called “*BAG-domain*“ (orange in Figure 1). However, this does not exclude the possibility that other regions of Hap46/BAG-1M and isoforms may also contribute to the interaction with hsp70 molecular chaperones. On the part of hsp70/hsc70, the ATP-binding domain is involved in the interaction, while the substrate binding domain remains available for further binding reactions [for reviews, *cf*. refs. 33,34]. This raises the possibility of multiple associations, and experiments with model proteins have in fact shown that trimeric complexes are easily formed [3,15,28,30,39]. Hsp70/hsc70 molecular chaperones as mediators of interactions also explain why very different bait proteins used in the original cloning experiments lead to the discovery of the same molecular sequence: hsc70 contained in these systems was able to serve as mediator. Interestingly, variations of the BAG-domain also exist in other proteins, which similarly mediate interactions with hsp70 chaperones; together they constitute the family of eukaryotic BAG proteins [38,40–42].

An interesting feature of hsp70 molecular chaperones is the structural flexibility of the two subdomains within the ATP-binding domain relative to each other. This was observed in experiments which studied the complex of the ATP-binding domain of the prokaryotic hsp70 protein DnaK with its regulator GrpE. X-ray crystallography disclosed contacts on both subdomains I and II with a significant opening of the cleft between them, as has been described clearly [37]. This readily explains why GrpE causes enhancement of the ADP/ATP exchange reaction of DnaK. Even though this movement of subdomains came to light by studies with the bacterial hsp70 protein DnaK, it is assumed to similarly occur in eukaryotic hsp70/hsc70 forms. Although structurally completely different, Hap46/BAG-1M and isoforms are functionally in a way eukaryotic counterparts of GrpE, however GrpE plays an essential role for the activity of DnaK in contrast to Hap46/BAG-1M and isoforms, whose effects on nucleotide exchange are more moderate. While GrpE binds asymmetrically as a dimer to DnaK, Hap46/BAG-1M or isoforms produce stoichiometric 1:1 complexes with eukaryotic hsp70 chaperones, even though hsp70s are normally much more abundant within cells. When the interaction between the BAG-domain and hsp70 was studied by X-ray analysis, NMR spectroscopy, and mutagenesis it was found that it forms a bundle of three α-helices that contact subdomain II of the ATP-binding domain [43,44]. Electrostatic interactions are of major importance in this interaction, whereby Arg308 (in the human Hap50/BAG-1L sequence) plays a major role, since exchange for alanine greatly reduced binding as well as *in vitro* activity; however other residues are also of importance. On the part of hsp70, the major portion of subdomain II suffices for binding, although the affinity is greatly reduced relative to the intact ATP-binding domain [45]. The techniques of phage display and of peptide scanning, however, resulted in several potential contact sites in two major regions, one on each subdomain I and II [46], suggesting that Hap46/BAG-1M and isoforms may use more than one contact with hsp70 chaperones for exerting their cellular effects. Binding of the BAG-domain probably favors an alternative structure of the ATP-binding domain that has a more open conformation [47], again reflecting flexibility of the structure.

With hsp70 molecular chaperones as major helpers in protein folding reactions in conjunction with hsp40, there immediately arose the question whether Hap46/BAG-1M and isoforms may affect this fundamental cellular activity in addition to the nucleotide exchange reaction. This was indeed observed in *in vitro* assays which employed various denatured proteins, in particular firefly luciferase and ß-galactosidase. While most reports describe inhibition of protein reactivation concomitant with reduced binding of misfolded proteins to the hsp70 molecular chaperone, positive effects on protein folding have also been observed [48–50]. This discrepancy is probably caused by details in experimental systems. Particularly in the presence of inorganic phosphate and at low concentrations of Hap46/BAG-1M protein renaturation was activated [50]; also the relative molar concentrations of hsp70 chaperone and Hap46/BAG-1M or isoform, as well as the type of isoform play important roles. Importantly, experiments with intact mammalian cells in culture also showed attenuation by overexpression of mouse BAG-1S of refolding denatured luciferase introduced into cells and thermally inactivated within them [51]. Thus Hap46/BAG-1M and isoforms have quite adequately been called *cochaperones*. This family of proteins, however, is but one of several known cochaperones which affect the protein folding and nucleotide exchange reactions of hsp70s, albeit in quite different ways. They certainly influence each others effects [see for example, refs. 28,52], so that a functional network exists that regulates the activities of hsp70 molecular chaperones.

## 4. Interactions with proteins independent of hsp70 molecular chaperones

In principle, Hap46/BAG-1M and isoforms might interact with various protein structures through mediation by hsp70/hsc70 molecular chaperones, as pointed out, or they may do so directly, *i.e.* independent of such mediation. In addition, Hap46/BAG-1M and Hap50/BAG-1L have the ability to bind to DNA, as discussed below. Of course, combinations between these types of interactions are expected to exist (see below). In any event, a multitude of bioactivities is produced by Hap46/BAG-1M and isoforms by such interactions.

**Figure 3.**
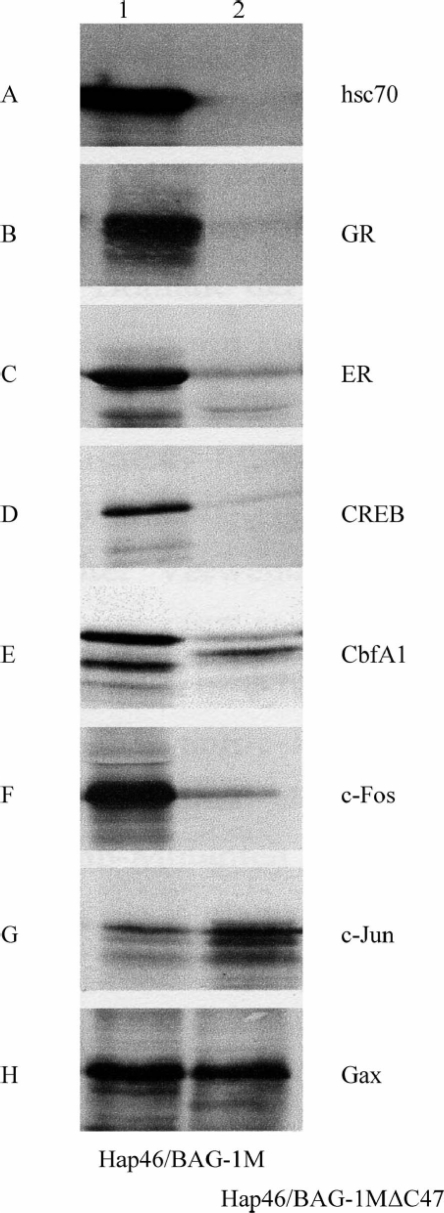
Formation of Hap46/BAG-1M complexes with several transcription factors. Transcription factors, as indicated, were synthesized as radiolabeled proteins by *in vitro* transcription/translation from recombinant constructs. They were then incubated in the presence of hsc70, contained in the reticulocyte lysate, with Sepharose matrices to which either Hap46/BAG-1M (lanes 1) or deletion variant Hap46/BAG-1MΔC47 (lanes 2) had been attached. After extensive washing, retained proteins were eluted and analyzed by electrophoresis in SDS-containing polyacrylamide gels and radioautography. Glucocorticoid receptor (GR); Estrogen receptor (ER). From ref. [53].

To check for the involvement of hsp70/hsc70 molecular chaperones, we compared the retention of *in vitro* synthesized and radiolabeled proteins on a matrix to which either full-length Hap46/BAG-1M or deletion variant Hap46/BAG-1MΔC47 had been coupled from which the carboxy terminal 47 amino acid residues, the BAG-domain, are missing [15]. As expected, hsc70 contained in large amounts in the reticulocyte lysate used for *in vitro* translation was not retained by the matrix containing the deletion variant (Figure 3 A). Moreover, this variant matrix did not bind any protein that requires mediation by hsc70 molecular chaperones, as is the case for several transcription factors which had been selected to be structurally different (Figures 3 B to F). Examples are members of the nuclear receptor superfamily, like the glucocorticoid receptor (GR) and the estrogen receptor (ER), as well as the cAMP response element binding protein CREB, the regulator of osteoblast differentiation CbfA1/Runx2, and c-Fos, a component of transcription factor AP-1. By contrast, c-Jun, the companion protein of c-Fos in AP-1, and Gax, a transcription factor involved in regulating cell viability, attached to roughly similar extents to both matrices (Figures 3 G and H). Apparently, these protein factors do not depend on any hsp70 molecular chaperone as binding mediator; c-Jun even appeared to interact somewhat better with the matrix devoid of hsc70 binding ability.

Protein kinase Raf-1 was the first example of a protein with the ability for direct and specific interaction with mouse BAG-1S, thereby causing activation of the kinase activity [26]. The binding site was mapped adjacent to and partially overlapping with that for hsp70 and point mutations within the BAG-domain which destroyed hsp70 binding activity were still able to activate the Raf-1 kinase [54]. Induction of hsp70 by cell stress caused downregulation of the kinase activity in mammalian cells. Thus competition of Raf-1 and hsp70 for binding to BAG-1S was proposed as molecular basis for the coordination of signals for cell growth and mitogenesis [54].

The retinoblastoma susceptibility protein Rb is another direct binding partner of Hap46/BAG-1M since it is able to interact with mutants defective in hsp70/hsc70 binding [55]. The complex of Rb and Hap46/BAG-1M is stable enough so that Hap46/BAG-1M is cotransported by Rb into the nucleus. The complex is disrupted by the human papilloma virus oncoprotein E7, an inhibitor of Rb protein interactions [55], thus emphasizing the specificity of complex formation. This modulation of subcellular localization of Hap46/BAG-1M has significant consequences for the survival of colorectal epithelial and tumor cells [56], suggesting a function of Rb as physiological regulator of Hap46/BAG-1M.

Hap50/BAG-1L was observed to stimulate the transactivation function of the androgen receptor, another member of the nuclear receptor superfamily, however, Hap46/BAG-1M did so only when forced into the nucleus [23]. While the carboxy terminal hsp70 binding BAG-domain mediates *in vitro* interaction, the amino terminal portion of Hap50/BAG-1L is required for nuclear localization and for eliciting the biological effect [23]. This is quite reasonable since both the ligand-activated androgen receptor as well as Hap50/BAG-1L or Hap46/BAG-1M need to be nuclearly localized in order to cooperate functionally. A similar argument may also apply to the interaction with both types of estrogen receptors, since Hap50/BAG-1L, but not the shorter isoforms, increased estrogen-dependent transcription in mammary carcinoma cells [57]. The cooperation with members of the nuclear receptor family, however, is not at all uniform, but rather complex [58]. Transactivation by the vitamin D receptor was also enhanced by Hap50/BAG-1L, but not by shorter non-nuclear forms, and binding again depended on the amino terminal part of the molecule. Interestingly, not only a variant from which the carboxy terminal BAG-domain had been deleted, but also overexpression of Hap50/BAG-1L itself inhibited vitamin D-dependent transactivation [59,60], suggesting a regulatory role of Hap50/BAG-1L in the action of vitamin D.

Interestingly, a variant of Hap50/BAG-1L has been isolated from a human T-cell cDNA library in a yeast two-hybrid screen. This turned out to be carboxy terminally truncated by 34 amino acids and harbors alterations within terminal residues [61]; as a consequence, it is unable to interact with hsp70 molecular chaperones. This variant is nevertheless able to associate with the tubulin-associating protein RP1. Thus RP1 is another direct interaction partner of Hap50/BAG-1L and most probably of other isoforms as well, thus linking these to the microtubule system. The functional consequences of this interaction for cellular mobility, cytokinesis and the cytoskeleton are certainly of interest.

## 5. Interaction with DNA

**Figure 4.**
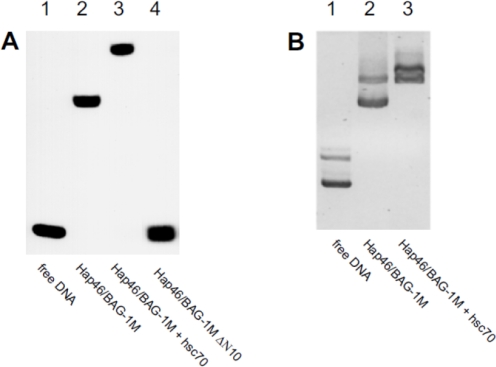
Binding of Hap46/BAG-1M to DNA. (A) A radiolabeled 283 basepair DNA fragment was either employed as such (lane 1) or used for electrophoretic mobility-shift assays with full-length Hap46/BAG-1M (lane 2), a deletion variant missing the amino terminal 10 residues (lane 4), or Hap46/BAG-1M plus hsc70 (lane 3). Detection was by autoradiography. From ref. [13]. (B) A circular plasmid of about 6 kilobases (as monomer and in dimeric form) was submitted to electrophoresis in 0.8% agarose gels either as such (lane 1), after incubation with Hap46/BAG-1M (lane 2) or with Hap46/BAG-1M plus hsc70 (lane 3). Detection of DNA was by staining with ethidium bromide. From ref. [15].

Although Hap46/BAG-1M is an acidic protein with a p*I* of 5.3, it nevertheless contains a prominent cluster of basic amino acids right next to the amino terminus [2,10] which is quite unique. This region was in fact found to be involved in *binding to DNA* (Figure 4 A, lane 4 *vs.* 2). The DNA may be linear (Figure 4 A) or circular (Figure 4 B), and neither the origin nor the size of the DNA matter, except that a minimum length is required [13,15]. Multiple copies of Hap46/BAG-1M can simultaneously associate with DNA, possibly in a cooperative manner, since the upshift of complexes upon gel electrophoresis occurs progressively [13,15].

In contrast to a previous report which suggested a specific binding site within the Cytomegalovirus promoter [62], we did not detect any such nucleotide sequence specificity when we investigated Hap46/BAG-1M with two series of overlapping oligonucleotides of about 130 and 240 base pairs which cover the entire early gene promoter of the Cytomegalovirus [15]. However, this does not exclude limited preferences for some as yet unidentified combinations of nucleotides. On the other hand, such non-specific DNA binding completely depends on the positively charged sequence close to the amino terminus of the polypeptide chain, which consists of three consecutive lysines and of three arginines, separated by a centrally located neutral residue (*cf.* legend to Figure 1). Mutation analysis established that both of these trimeric clusters are required for the DNA binding ability of Hap46/BAG-1M [13,16,62], but the exact spacing between them does not matter. This non-specific DNA binding motif is involved in down-regulation of glucocorticoid receptor-mediated transactivation by Hap46/BAG-1M [16,63], even though the mechanism of this effect is not clear. In Hap50/BAG-1L the same basic peptide region (red in Figure 1) is available for interaction with DNA, but the polypeptide chain is extended further amino terminally and contains additional basic residues, which may be involved as well.

It is important to point out that both major interaction regions located close to the ends of the amino acid sequences of Hap46/BAG-1M and Hap50/BAG-1L, *i.e.* for DNA binding and for interaction with hsp70 molecular chaperones, are available for respective binding reactions at the same time. Thus trimeric complexes of the type DNA•Hap46/BAG-1M•hsc70 can easily be formed [13,15], as shown in Figure 4 (A and B, lanes 3). In these complexes the substrate-binding domain of hsc70 is still available for additional interactions with other proteins, for example transcription factors.

## 6. Effect of Hap46/BAG-1M and Hap50/BAG-1L on transcription

When we realized that Hap46/BAG-1M has the ability to interact with DNA, there immediately arose the idea that it might affect transcriptional activities, in particular since thermal stress causes a significant portion of Hap46/BAG-1M to translocate from the cytoplasm to the cell nucleus (*cf.* Figure 2, B *vs.* A). By contrast, Hap50/BAG-1L (Figure 2 C) is mainly localized in the nucleus under normal cell culture conditions [6,7,14,23], a fact that immediately points to a function within the nucleus.

**Figure 5.**
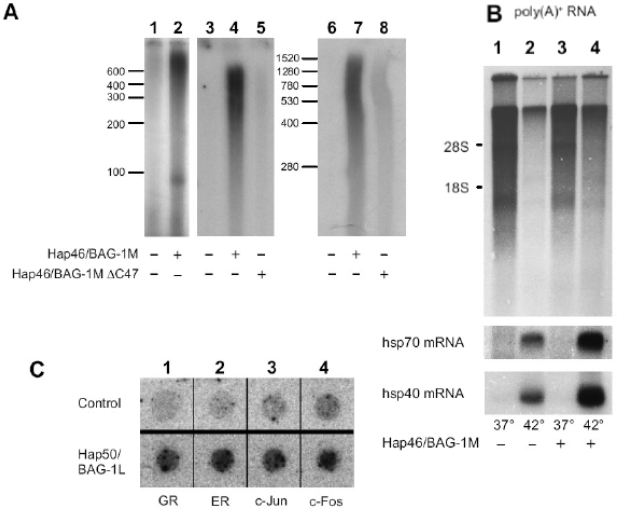
Stimulation of transcription by Hap46/BAG-1M and Hap50/BAG-1L. (A) *In vitro* transcription assays were carried out with HeLa nuclear extracts using either plasmid DNA (lanes 1 and 2), linearized prokaryotic DNA without a promoter (lanes 3 to 5), or eukaryotic, linear DNA without a promoter (lanes 6 to 8). Hap46/BAG-1M (lanes 2, 4, 7) or the carboxy terminally deletion variant Hap46/BAG-1M C47 (lanes 5 and 8) were added, as indicated. Newly synthesized radiolabeled RNA was analyzed by gel electrophoresis and autoradiography. Positions of RNA markers are shown along the margins. From ref. [15]. (B) DU145 cells were transfected with an expression vector coding for Hap46/BAG-1M (lanes 3 and 4) or not (lanes 1 and 2). As indicated, cells were then submitted to heat treatment at 42°C for 2 h. Newly synthesized radiolabeled poly(A)^+^RNA was analyzed by gel electrophoresis and autoradiography (upper panel). The positions of 18 S and 28 S rRNAs are shown along the margin. The same filters were subsequently used for Northern hybridization with radiolabeled cDNAs encoding hsp70 or hsp40 (lower panels). From ref. [13]. (C) HeLa cells were transfected with an expression vector coding for Hap50/BAG-1L (lower panel) or not (upper panel) and used for nuclear runoff transcription assays. Newly synthesized radiolabeled RNAs were probed with dot-blotted cDNAs encoding the glucocorticoid receptor (GR), the estrogen receptor (ER), c-Jun, or c-Fos, as indicated. Detection was by autoradiography. From ref.[14].

Figure 5 A shows *in vitro* transcription experiments using HeLa nuclear extracts. Addition of Hap46/BAG-1M to these assays resulted in roughly a 10-fold *stimulation of transcription* from various templates [13,15]. It did not matter whether eukaryotic or prokaryotic DNA was used as template and whether or not it contained promoter elements. The fact that the transcripts have no distinct length suggests random initiation of transcription and termination at the end of linearized DNA fragments. Involvement of the amino terminal DNA binding region in the effect on transcription became evident from the observation that deletion of the 10 amino terminal residues from Hap46/BAG-1M resulted in complete loss of stimulation [13].

These *in vitro* experiments were then complemented by studies with intact human cells transfected with an expression vector for Hap46/BAG-1M. Upon heat treatment of these cells which results in nuclear translocation (*cf.* Figure 2 B), overexpression of Hap46/BAG-1M largely compensated the drastic shut-down of cellular transcription which normally occurs following heat stress (Figure 5 B upper panel, lane 4 *vs*. 2). Interestingly, induction of heat shock proteins hsp70 and hsp40 was further stimulated by Hap46/BAG-1M (Figure 5 B lower panels, lane 4 *vs*. 2). Upon overexpressing Hap50/BAG-1L in a similar cell system, we again observed increased transcriptional activities (*cf.* Figure 5 C). The expression of several endogenous genes which are under cellular regulation, like those coding for c-Jun, c-Fos, or the receptors for glucocorticoids or estrogens were stimulated, while that for actin, for example, was not enhanced [14].

## 7. Molecular model for the effects on transcription

**Figure 6.**
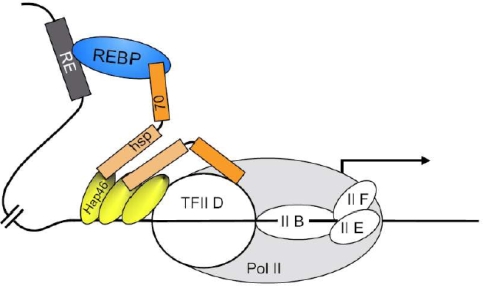
Molecular model for the effects of Hap46/BAG-1M on transcription. The transcription initiation complex is shown in a simplified version. Arbitrarily, three molecules of Hap46/BAG-1M are depicted in association with DNA and are supposed to establish contacts *via* hsp70 molecular chaperones. Pol II: RNA polymerase II; TFII: basal transcription factor II; RE: response element on DNA; REBP: specific response element-binding protein, for example a liganded and activated steroid hormone receptor. From ref.[64].

Taking the above observations into consideration, it appears that the effects of Hap46/BAG-1M and Hap50/BAG-1L on transcription are of major physiological relevance. This activity of Hap46/BAG-1M is discussed here in terms of a *molecular model* that is depicted in Figure 6. Two prominent features of this protein are taken into account: DNA binding ability and the potential to interact with a variety of transcription factors either by mediation through hsp70 molecular chaperones or directly (*cf.* Fig 3). Several molecules of Hap46/BAG-1M might bind simultaneously to a stretch of DNA within a region of transcriptionally active chromatin and establish contacts to the transcriptional apparatus itself, as well as to transcripion factors attached to DNA *in cis*, but possibly at distant locations. In this way, Hap46/BAG-1M may help to assemble various cofactors into functional complexes, eventually causing increased expression of the respective gene. A transcription factor attached to Hap46/BAG-1M, as shown in Figure 6, may for example, be identical to a steroid hormone receptor, which itself is bound to a specific response element on the DNA in some distance to the gene to be transcribed.

The same considerations similarly apply to the large isoform Hap50/BAG-1L. Interestingly, Hap50/BAG-1L was found to increase estrogen-dependent transcription in mammary carcinoma cells and thus turns out as important determinant of estrogen function [57]. Of course, several transcription factors might come into contact through multiple molecules of Hap46/BAG-1M or Hap50/BAG-1L attached to DNA. Chromatin immunoprecipitation studies in which Hap50/BAG-1L, the molecular chaperone hsp70, and the androgen receptor were found targeted to the relevant response element [65] support the above notion and clearly favor the nucleation of transcriptional regulatory complexes, as proposed here.

The effects of Hap46/BAG-1M and Hap50/BAG-1L on transcription, however, do not necessarily need to be positive, and in case of steroid hormone receptors as transcriptional regulators, the responses are not at all uniform [58]. The inhibitory activity of Hap46/BAG-1M on glucocorticoid receptor-mediated transcription was found to depend on the amino terminal DNA binding region, while Hap33/BAG-1S in contrast, did not elicit a similar effect. In addition, an intact carboxy terminal domain for interaction with hsp70 molecular chaperones was also found to be required [16,63]. This implies that both functional domains close to the ends of the Hap46/BAG-1M sequence need to be present in *cis*.

The molecular model of Figure 6 suggests several details which still require experimental elucidation. Most importantly, it will be of interest which genes or groups of genes are affected by Hap46/BAG-1M and by Hap50/BAG-1L. In addition, it will be interesting to find out how various transcription factors cooperate molecularly with Hap46/BAG-1M and Hap50/BAG-1L to bring about specific effects. In addition to the tumor suppressor protein Rb, c-Jun, and Gax, other transcription factors may interact directly with the transcription stimulating protein Hap46/BAG-1M, *i.e.* independent of hsp70 molecular chaperones. Transcription factor AP-1 is of particular interest in the present context since one component, c-Fos requires mediation by an hsp70 chaperone, while the other, c-Jun is able to interact directly with Hap46/BAG-1M (*cf.* Figures 3 F and G). It will be interesting to see which region of Hap46/BAG-1M is involved in this direct interaction and whether it overlaps with some of the other binding sites. The above difference between c-Fos and c-Jun is of significance as they form functionally active heterodimers in AP-1, and the DNA binding of heteromeric AP-1 was found to be selectively modified by hsp70 heat shock proteins relative to homodimeric c-Jun [66]. This supports the notion that the DNA binding proteins Hap46/BAG-1M and AP-1 together with hsp70 molecular chaperones cooperate in forming a network of protein-protein and protein-DNA interactions which may be involved in targeting and activating the basal transcription machinery.

While addition of Hap46/BAG-1M to the *in vitro* transcription system clearly caused a rather large positive effect (*cf.* Figure 5 A, lanes 2, 4, and 7 *vs.* 1, 3, and 6), it came as a surprise that the carboxy terminal deletion variant Hap46/BAG-1MΔC47 also produced some, albeit moderate stimulation [15], as shown in Figure 5 A (lanes 5 and 8 *vs.* 3 and 6). This points to involvement of hsp70 molecular chaperones and suggests that they as well play an important role in transcription. Remodeling of transcriptional regulatory complexes by molecular chaperones has indeed been pointed out before [67,68]. It is of significance in this context that newly synthesized hsp70 in response to cellular stress readily becomes nuclearly localized [69]. Since Hap46/BAG-1M also accumulates in the cell nucleus following heat shock, cooperation between these proteins to affect transcription, as proposed in the model of Figure 6, appears reasonable for regulating cellular activities.

## 8. Concluding remarks

In contrast to Hap46/BAG-1M and Hap50/BAG-1L, the prominently expressed short isoform Hap33/BAG-1S which is inert towards DNA appears most suitable for affecting protein folding and/or degradation. Since multiple biochemical reactions possibly cooperate in eliciting complex cellular responses, it is conceivable that modulation of transcription by Hap50/BAG-1L or Hap46/BAG-1M as well as the effects of Hap33/BAG-1S on refolding or degradation of misfolded proteins contribute to a cells defense against apoptosis. It will be important to clarify the individual shares of these cellular reactions. Depending on its prevalent intracellular localization, Hap46/BAG-1M may even serve a dual purpose: transcriptional modulation within the nucleus and control of protein functionality in the cytoplasm, thus providing a molecular link between such diverse activities and helping the cell to survive under adverse conditions. The pleiotropic activities of Hap46/BAG-1M and isoforms within the nucleus may also include control of protein quality. In any event, the intracellular localization of Hap46/BAG-1M and isoforms is rather dynamic, with relocalization possibly depending on specific signals as well as on the distribution of interacting protein factors, most importantly hsp70 chaperones. Thus, MCF7 human mammary carcinoma cells under heat stress were described to relocalize Hap33/BAG-1S from the cytoplasm to the nucleus [21] concomitant with nuclear accumulation of hsp70, thereby preventing stress-induced growth inhibition. Recruitment of Hap46/BAG-1M to the nucleus by activated glucocorticoid receptors has also been reported [17], but it is not clear whether this occurs in conjunction with hsp70 molecular chaperones. As mentioned above, nuclear transport of Hap46/BAG-1M may as well occur together with the retinoblastoma protein Rb [55,56]. Nevertheless, the regulation of expression of different isoforms needs to be investigated, which may depend, at least to some extent, on cap-independent initiation of translation at internal ribosome entry sites [9,70]. This is of interest since stage- and site-specific expression of isoforms has been observed during early murine development [71]. Moreover, the involvement of these proteins in the regulation of cell differentiation has been observed in several systems [72,73].

A very exciting case of regulation was discovered upon disrupting the *Bag1* gene in mice [74]. Homozygous Bag1^−/−^ embryos were severely growth-retarded, died before term, and showed multiple developmental defects. Particularly, cell survival within the nervous and hematopoietic systems was affected, again emphasizing the anti-apoptotic potential of BAG-1 proteins. Even though the protein kinases Raf/ERK and Akt were *per se* not defective in these mutant animals, the protein phosphorylation pattern was affected with loss of phosphorylation of the regulatory protein Bad at Ser136, which may be caused by changes in intracellular distribution and targeting [74]. These observations suggest that at least one of the products of the *Bag1* gene functions as mediator of extracellular survival signals which normally prevent apoptosis in certain stem cells and point to the exciting fact that members of the Hap46/BAG-1M and Hap50/BAG-1L protein family fulfill some vital function within the mammalian organism. It is of interest to find out to which extent hsp70 molecular chaperones are involved in the above cellular effects. The growth of neurites has been studied in cell culture using rat phaeochromocytoma cells PC12 primed with NGF [75]. It was found that overexpression of the small isoform BAG-1S interferes with neurite extension and that interaction with an hsp70 molecular chaperone plays a crucial role in this effect. By contrast, the large isoform Hap50/BAG-1L did not inhibit neurite outgrowth in such neurotrophin-treated cells. Significant differences in intracellular localization of these isoforms may be responsible for such functional differences.

Obviously, Hap46/BAG-1M, Hap50/BAG-1L and isoforms play an important role in modulating cell survival. This is of particular importance under cellular stress, in neoplastic cell conditions and even under anti-tumor therapy. Cells highly expressing the *Bag1* gene are probably selected for by cytotoxic treatments. Thus, multidrug-resistant cells of decreased sensitivity towards apoptosis were observed to express Hap46/BAG-1M and Hap33/BAG-1S at rather high levels [76].

Hap50/BAG-1L and Hap46/BAG-1M may be regarded as *pro-survival proteins*, and thus became markers for the aggressiveness of various cancers, in particular of mammary carcinomas; they are predictive factors for the *clinical prognosis* of patients. Such correlations have been observed in many clinical and cell culture studies [for example, refs. 7,77–85]. Hap50/BAG-1L in particular is often expressed at rather high levels in tumors and metastases as compared to normal cells. It is of interest in this context that expression of the *Bag1* gene is enhanced by tumor-derived p53 mutants [11] and that methylation reactions play an important role in its expression [12]. Consequently, Hap50/BAG-1L and Hap46/BAG-1M may serve in the future as molecular targets for therapeutic approaches, as has been discussed [83,86]. Indeed, the immunosuppressant rapamycin [87,88] and the protein kinase inhibitors flavopiridol and 7-hydroxy-staurosporine [89] are known to downregulate the expression of the *Bag1* gene, thereby affecting apoptotic cell responses. By contrast, interleukin-2 as well as other cell survival signals were found to upregulate the expression of the *Bag1* gene in different cell types [9,87]. Retinoic acid-induced apoptosis in breast cancer cells in culture is inhibited by the interaction of Hap33/BAG-1S with retinoic acid receptors and interference with their ability to bind to specific DNA response elements [25], while the apoptotic activity of *N*-(4-hydroxyphenyl)retinamide, used for chemoprevention and treatment of various cancers, was enhanced by overexpression of Hap50/BAG-1L or shorter isoforms in a cervical carcinoma cell line [21]. On the other hand, HeLa cells in which the levels of Hap50/BAG-1L and Hap33/BAG-1S were down-regulated by RNA interference, had lower growth rates and became less sensitive to several anti-cancer drugs [90]. Also, overexpression of the *Bag1* gene in mammary carcinomas may limit the efficacy of PPARγ agonists, like 15-deoxy-PGJ_2_, as apoptosis-inducing agents [91]. This brief discussion already points to the complexity of the problem and certainly shows that much more basic information is needed. Nevertheless, some of the above mentioned observations raise the hope that it will be possible in the future to manipulate the cellular levels of Hap50/BAG-1L and Hap46/BAG-1M. It could thus be highly beneficial for the *in situ* treatment of several cancer types if the levels of these isoforms within tumor cells were lowered by specific agents. This may then occur either in conjunction with hsp70 chaperone activities or independent of them.
